# Malignant Pertussis With COVID-19 Coinfection in an Unvaccinated Infant: A Case Report in the Context of Vaccine Hesitancy

**DOI:** 10.7759/cureus.109673

**Published:** 2026-05-26

**Authors:** Mariam M Elmakkawy, Thong Pui Ling, Mohd Azri Zainal Abidin

**Affiliations:** 1 Pediatrics, Hospital Sultan Abdul Aziz Shah, University Putra Malaysia, Selangor, MYS

**Keywords:** bordetella pertussis, coinfection in covid-19, covid-19, malignant pertussis, respiratory infection, sars-cov-2 (severe acute respiratory syndrome coronavirus-2), vaccine hesitancy

## Abstract

Pertussis remains a major cause of morbidity in infants worldwide despite widespread vaccination. Coinfection with respiratory viruses such as severe acute respiratory syndrome coronavirus 2 (SARS-CoV-2) may exacerbate disease severity and complicate management. We report a case of malignant pertussis in a one-year and five-month-old boy complicated by a concurrent coronavirus disease 2019 (COVID-19) infection. The child presented with prolonged paroxysmal cough, leukocytosis, and respiratory distress requiring non-invasive ventilation. *Bordetella pertussis* was confirmed by nasopharyngeal aspirate polymerase chain reaction (PCR). The patient also tested positive for SARS-CoV-2. He was managed with supportive care and dual antibiotic therapy, with gradual clinical improvement and normalization of white cell count. This case highlights the diagnostic and therapeutic challenges of pertussis in the setting of co-infection and delayed immunization.

## Introduction

Pertussis, caused by *Bordetella pertussis*, remains a major cause of morbidity and mortality among infants worldwide, despite the global availability of effective vaccination programs [1]. The infection is particularly severe in young infants who are unvaccinated or under-immunized, as maternal antibodies provide limited protection and vaccine-induced immunity wanes over time [2,3]. According to the World Health Organization, periodic resurgences of pertussis continue to occur even in highly vaccinated populations, largely attributed to incomplete immunization coverage, waning immunity, and pathogen adaptation [3].

Coinfection with respiratory viruses, including severe acute respiratory syndrome coronavirus 2 (SARS-CoV-2), can further exacerbate disease severity by impairing ciliary function, damaging airway epithelium, and disrupting immune homeostasis [4]. Reports of *Bordetella pertussis* and SARS-CoV-2 coinfection remain rare but clinically significant, as overlapping symptoms such as persistent cough and respiratory distress may obscure diagnosis and delay appropriate management [5]. During the COVID-19 pandemic, disruptions in routine childhood immunization programs, coupled with growing vaccine hesitancy, have widened immunity gaps and increased susceptibility to vaccine-preventable infections [3].

Severe or “malignant” pertussis represents the most critical form of the disease, characterized by marked hyperleukocytosis, pulmonary hypertension, and respiratory failure that often necessitates intensive care admission. Mortality rates in malignant pertussis can exceed 75% among critically ill infants, primarily due to cardiorespiratory failure secondary to pertussis toxin-mediated vascular injury [6]. The disease pathophysiology involves toxin-induced lymphocytosis, endothelial damage, and microthrombotic changes that contribute to pulmonary hypertension and right ventricular strain [7]. Early recognition of these complications is vital, as echocardiographic evidence of pulmonary hypertension is a strong predictor of poor outcomes [8].

Here, we report a case of malignant pertussis complicated by concurrent SARS-CoV-2 infection in an unvaccinated infant. The case highlights the diagnostic complexity, therapeutic considerations, and public health implications of dual infection, underscoring the importance of vaccine confidence and adherence to immunization schedules as essential strategies for disease prevention.

## Case presentation

An unvaccinated Malay male infant, aged one year and five months, with a weight of 9.6 kg (5th percentile), height of 81 cm (50th percentile), and head circumference of 47 cm (25th-50th percentile), presented with a three-week history of persistent cough. The illness initially manifested with mild cough and rhinorrhoea, progressing to paroxysmal bouts associated with post-tussive vomiting. Two weeks after onset, he developed fever, tachypnoea, and poor oral intake. A rapid antigen test for COVID-19 was positive; however, the family initially declined admission due to insurance constraints. Such a clinical presentation is consistent with classical pertussis in infants [1,2]. Polymerase chain reaction (PCR) remains the diagnostic gold standard for confirmation [3]. Coinfection with SARS-CoV-2, though rarely reported, has been associated with exacerbated respiratory distress [4]. The use of non-invasive ventilation aligns with current best practice for managing moderate respiratory compromise [5].

**Figure 1:**
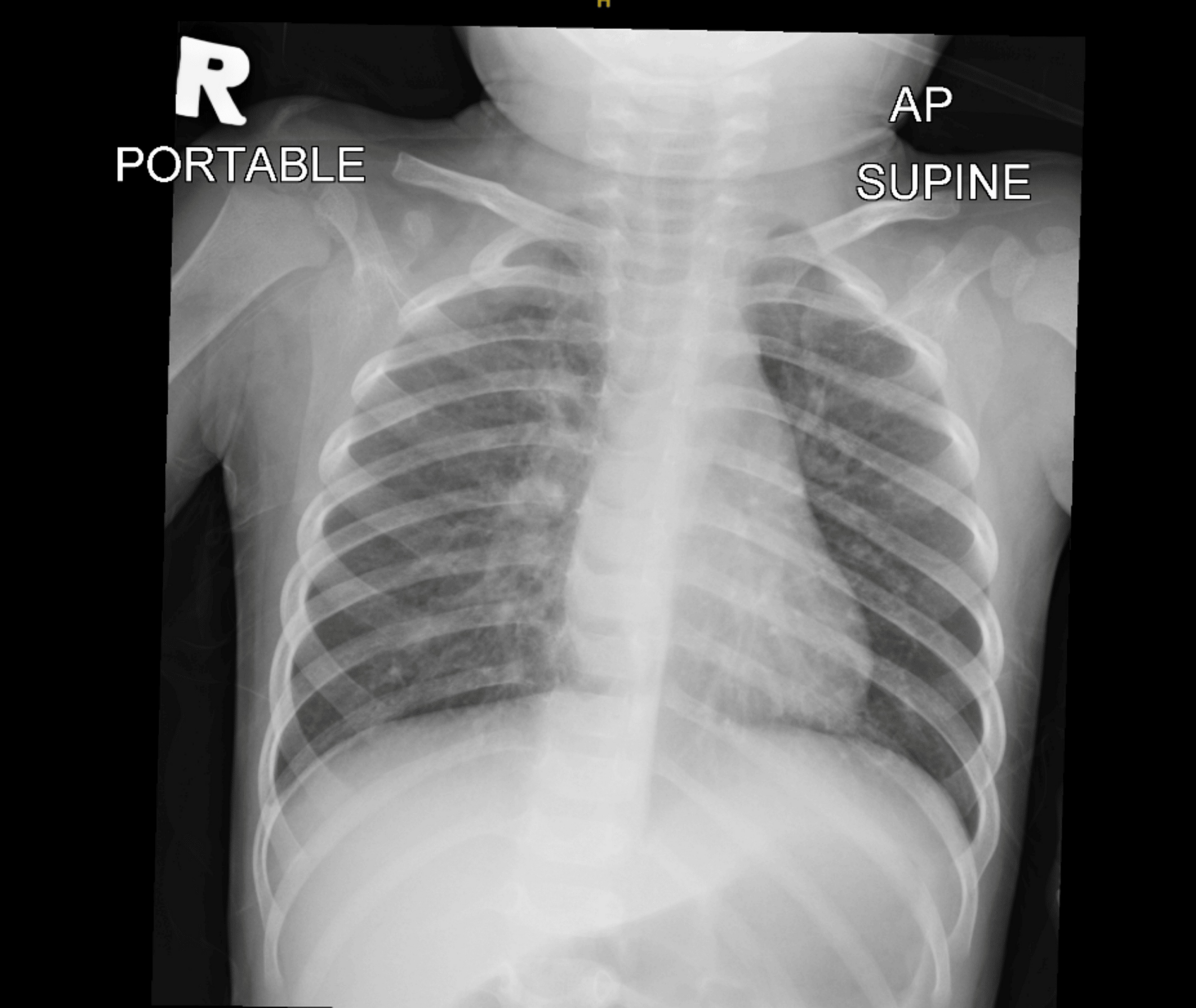
Chest radiograph on admission. Portable anteroposterior (AP) supine chest radiograph of an infant showing hyperinflated lungs with flattened diaphragms and bilateral perihilar interstitial infiltrates, more prominent in the right lower and left perihilar zones. The cardiac silhouette is within normal limits. No focal consolidation, pleural effusion, or pneumothorax is observed. These findings are consistent with viral-induced pneumonitis and airway inflammation secondary to *Bordetella pertussis *infection, with possible contribution from concurrent SARS-CoV-2 infection.

Upon admission to our institution, the patient appeared lethargic and tachypneic. A chest radiograph was done showing hyperinflated lungs with flattened diaphragms and bilateral perihilar interstitial infiltrates, more prominent in the right lower and left perihilar zones. The cardiac silhouette is within normal limits. No focal consolidation, pleural effusion, or pneumothorax is observed (Figure 1).

He was initiated on bilevel positive airway pressure (BiPAP) with inspiratory positive airway pressure (IPAP) of 12 cm H_2_O, expiratory positive airway pressure (EPAP) of 6 cm H_2_O, and fraction of inspired oxygen (FiO_2_) of 30%. He remained hemodynamically stable without apnoea or seizures throughout hospitalization. 

Initial investigations revealed hyperleukocytosis (total white cell count (TWC) 57.28 × 10^9^/L), hemoglobin 11.2 g/dL, and platelet count 535 × 10^9^/L. C-reactive protein (CRP) was mildly elevated (8.2 mg/L), while the renal profile was within normal limits.

Serum albumin was low (30 g/L), suggestive of mild hypoalbuminemia secondary to inflammation or nutritional compromise, while total protein remained within the normal range. Liver enzyme levels (aspartate aminotransferase 35 U/L, alanine aminotransferase 13 U/L, alkaline phosphatase 177 U/L) were normal, excluding hepatic involvement. Peripheral blood smear demonstrated marked lymphocytosis without blasts, supporting a reactive rather than malignant process.

These findings are characteristic of malignant pertussis, particularly the profound leukocytosis and lymphocytosis with normal inflammatory markers, and are summarized in Table 1.

**Table 1:** Laboratory findings showing marked lymphocytosis and leukocytosis typical of malignant pertussis.

Test name (unit)	Observed value	Normal range
White blood cell count (×10^9^/L)	57.3	6.0–17.0
Neutrophils (%)	22	40–75
Lymphocytes (%)	73	20–40
Absolute lymphocyte count (×10^9^/L)	41.4	3.5–11.0
Hemoglobin (g/dL)	11.2	11.1–14.1
Mean corpuscular volume, MCV (fL)	66	72–84
Mean corpuscular hemoglobin, MCH (pg)	29.6	32-36
Platelet count (×10^9^/L)	535	200–550
C-reactive protein (mg/L)	8.2	<5.0
Urea (mmol/L)	2.8	2.7–8.1
Creatinine (µmol/L)	22	21–36
Sodium (mmol/L)	135	135–145
Potassium (mmol/L)	3.8	3.5–5.0
Calcium, total (mmol/L)	2.26	2.25–2.75
Magnesium (mmol/L)	0.92	0.70–0.95
Albumin (g/L)	30	38–54
Total protein (g/L)	60	60–80
Aspartate transaminase, AST (U/L)	35	10–50
Alanine transaminase, ALT (U/L)	13	10–45
Alkaline phosphatase, ALP (U/L)	177	145–420

**Figure 2:**
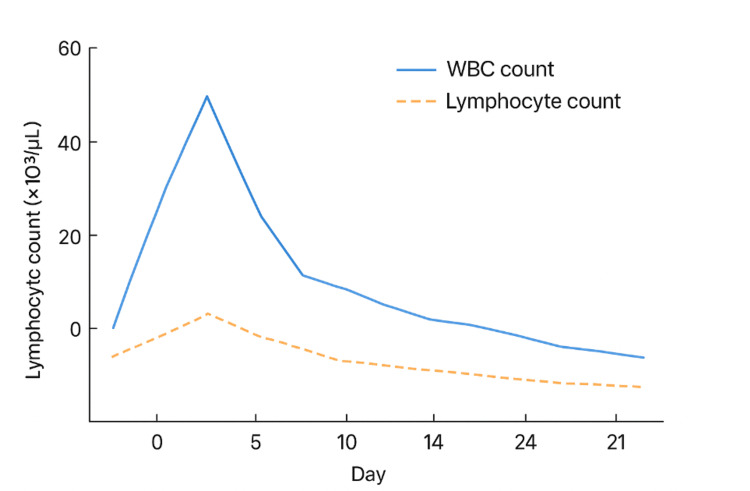
Leukocyte and lymphocyte trends during hospitalization. The line graph demonstrates the changes in white blood cell (WBC) and lymphocyte counts over 24 days of illness. A peak leukocytosis exceeding 50 × 10³/µL occurred on Day 5, followed by a gradual decline corresponding to the patient’s clinical improvement.

Echocardiography demonstrated normal cardiac function with no evidence of pulmonary hypertension. Nasopharyngeal aspirate PCR confirmed *Bordetella pertussis* infection, and SARS-CoV-2 PCR remained positive (COVID-19 Category 4). The patient received intravenous azithromycin and amoxicillin-clavulanate for 72 hours and was later transitioned to oral azithromycin and oral amoxicillin-clavulanate to complete a 10-day course. Serial monitoring showed a gradual decline in leucocytes: 57.3 → 45.9 → 35 → 33.4 → 35 → 25.37 → 21.37 → 11 × 10^9^/L (Figure 2).

Platelet count and CRP returned to normal. Respiratory support was gradually weaned from BiPAP (one week) to continuous positive airway pressure (two days), followed by low-flow nasal oxygen and finally room air. No cardiovascular complications were observed. At discharge, the patient was afebrile, feeding well, and maintaining adequate oxygen saturation on room air. Following counselling, the previously vaccine-hesitant parents agreed to initiate a catch-up immunization schedule.

**Figure 3:**
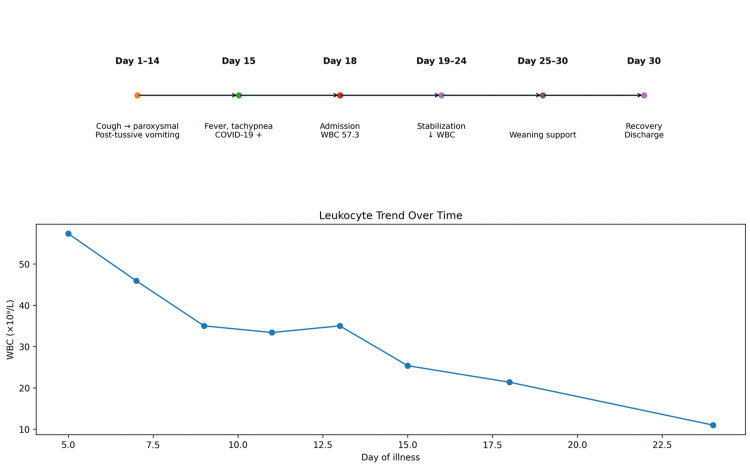
Clinical timeline and leukocyte trend.

## Discussion

Pertussis, or whooping cough, is a contagious respiratory infection caused by *Bordetella pertussis*. Although vaccination has greatly reduced its incidence, the disease persists globally, particularly in infants who are unvaccinated or under-immunized. Malignant pertussis represents the most severe form, characterized by extreme leukocytosis, pulmonary hypertension, and potential cardiovascular collapse. Mortality rates can exceed 75 % in critically ill infants. Severe forms, referred to as malignant pertussis, have been well documented [6,7]. The associated leukocytosis and risk of pulmonary hypertension are characteristic features [8]. The underlying mechanism involves pertussis toxin-induced lymphocytosis leading to hyperviscosity and microvascular compromise [9]. Macrolide therapy, such as azithromycin, remains the mainstay of treatment [1,3]. For cases with severe leukocytosis, exchange transfusion or leukapheresis has been reported to improve outcomes [6]. COVID-19 coinfection may worsen inflammation and delay diagnosis due to overlapping symptoms [4,10].

The pathogenesis of malignant pertussis involves pertussis toxin-induced lymphocytosis and pulmonary vascular injury. Pertussis toxin impairs lymphocyte migration, leading to massive accumulation of circulating lymphocytes and resultant hyperviscosity. Elevated pulmonary vascular resistance due to endothelial injury and microthrombosis may culminate in right-sided heart failure. Early echocardiographic evaluation is essential, as the appearance of pulmonary hypertension correlates strongly with mortality [8].

Our patient, despite peak TWC > 50 × 10^9^/L, showed no echocardiographic evidence of pulmonary hypertension, possibly reflecting early recognition, optimal ventilatory support, and effective reduction in leukocytosis.

Co-infection with SARS-CoV-2 in this case adds a unique dimension. Viral co-infections can exacerbate pertussis severity by damaging ciliated epithelium and altering immune responses, facilitating secondary bacterial adherence [4,10]. The COVID-19 pandemic also posed diagnostic challenges because both infections may present with prolonged cough and respiratory distress. In this child, the confirmation of *Bordetella pertussis* by PCR despite concurrent SARS-CoV-2 positivity underscores the importance of maintaining broad diagnostic consideration when evaluating protracted coughs in infants, particularly in the post-pandemic era [3,4].

Management of malignant pertussis centers on macrolide therapy to eradicate *Bordetella pertussis* and meticulous supportive care to manage toxin-mediated complications. Azithromycin remains the preferred macrolide because of its favorable safety profile and once-daily dosing [1,3]. However, antibiotic therapy alone does not immediately reverse the effects of pertussis toxin; thus, respiratory and circulatory support are pivotal. For severe leukocytosis with cardiorespiratory compromise, leukoreduction or exchange transfusion has been reported to improve outcomes, and Extracorporeal Membrane Oxygenation (ECMO) may be considered in refractory pulmonary hypertension. In our patient, hyperleukocytosis resolved gradually without invasive intervention, emphasizing that aggressive supportive management may suffice in selected cases [6-8].

A key contextual factor was vaccine hesitancy. The patient was under-immunized, leaving him susceptible to pertussis infection. Vaccine hesitancy, defined as the delay or refusal of vaccination despite availability, remains a global health threat. In Malaysia and many other countries, sporadic outbreaks of pertussis have been attributed to declining vaccine uptake and waning immunity. Addressing vaccine hesitancy through targeted education, clear communication of vaccine benefits, and building parental trust is essential to prevent similar cases. Notably, following counselling, the parents agreed to resume catch-up immunization.

From an epidemiologic perspective, the COVID-19 pandemic disrupted routine childhood immunization schedules worldwide, creating immunity gaps that may facilitate pertussis resurgence. Moreover, overlapping symptoms between COVID-19 and pertussis can delay specific diagnosis, particularly in under-immunized children. Therefore, clinicians should maintain vigilance and include *Bordetella pertussis* in the differential diagnosis of prolonged cough even when viral infection is confirmed.

In conclusion, this case typifies malignant pertussis complicated by SARS-CoV-2 co-infection in an incompletely immunized infant. The child recovered without pulmonary hypertension through early diagnosis, appropriate macrolide therapy, and non-invasive ventilatory support. It underscores the critical importance of vaccination, timely recognition, and multidisciplinary care in improving outcomes of this rare but life-threatening condition.

## Conclusions

This case underscores the importance of early recognition and comprehensive management of malignant pertussis, particularly when complicated by concurrent viral infections such as SARS-CoV-2. Coinfection may exacerbate disease severity through synergistic immune dysregulation and respiratory compromise. Early identification of characteristic hematologic patterns, including extreme leukocytosis and lymphocytosis, is vital for timely intervention.

Integration of molecular diagnostics, vigilant supportive care, and a multidisciplinary team approach can significantly improve outcomes in pediatric patients presenting with severe pertussis. Heightened clinician awareness of potential pertussis-COVID-19 coinfection is essential to prevent diagnostic delays, optimize management strategies, and reinforce the importance of routine childhood vaccination as a key preventive measure.
